# Modulating the antioxidant system by exogenous 2-(3,4-dichlorophenoxy) triethylamine in maize seedlings exposed to polyethylene glycol-simulated drought stress

**DOI:** 10.1371/journal.pone.0203626

**Published:** 2018-09-05

**Authors:** Tenglong Xie, Wanrong Gu, Liguo Zhang, Lijie Li, Danyang Qu, Caifeng Li, Yao Meng, Jing Li, Shi Wei, Wenhua Li

**Affiliations:** 1 College of Agriculture, Northeast Agricultural University, Harbin, P.R. China; 2 Maize Research Institute, Heilongjiang Academy of Agricultural Sciences, Harbin, P.R. China; 3 Heilongjiang Academy of Land Reclamation Sciences, Harbin, P.R. China; Estacion Experimental del Zaidin, SPAIN

## Abstract

Maize (*Zea mays* L.), an important agricultural crop, suffers from drought stress frequently during its growth period, thus leading to a decline in yield. 2-(3,4-Dichlorophenoxy) triethylamine (DCPTA) regulates many aspects of plant development; however, its effects on crop stress tolerance are poorly understood. We pre-treated maize seedlings by adding DCPTA to a hydroponic solution and then subjected the seedlings to a drought condition [15% polyethylene glycol (PEG)-6000 treatment]. The activities of superoxide dismutase (SOD), peroxidase (POD), ascorbate peroxidase (APX), and glutathione reductase (GR) were enhanced under drought stress and further enhanced by the DCPTA application. The activities of monodehydroascorbate reductase (MDHAR), dehydroascorbate reductase (DHAR) and catalase (CAT) declined continuously under drought stress; however, the activities partially recovered with DCPTA application. Up-regulation of the activities and transcript levels of APX, GR, MDHAR and DHAR in the DCPTA treatments contributed to the increases in ascorbate (AsA) and glutathione (GSH) levels and inhibited the increased generation rate of superoxide anion radicals (O_2_·^−^), the contents of hydrogen peroxide (H_2_O_2_) and malondialdehyde (MDA), and the electrolyte leakage (EL) induced by drought. These results suggest that the enhanced antioxidant capacity induced by DCPTA application may represent an efficient mechanism for increasing the drought stress tolerance of maize seedlings.

## Introduction

Reactive oxygen species (ROS), the by-products of aerobic metabolism, are continuously produced in plants and efficiently eliminated by plant antioxidant defence mechanisms under non-stress conditions [1]. However, drought stress inevitably alters the critical balance between the generation and scavenging of ROS, resulting in excessive levels of ROS [2]. These levels can damage biological membrane systems and macromolecules, resulting in the interruption of normal metabolism and thereby leading to the inhibition of plant growth [3].

To maintain ROS at non-toxic levels under stress conditions, plants have evolved an effective antioxidant defence system that involves a wide range of non-enzymatic and enzymatic antioxidants [4]. Superoxide radicals (O_2_·^−^) are converted into oxygen (O_2_) and hydrogen peroxide (H_2_O_2_) by superoxide dismutase (SOD) as the first step in ROS scavenging [5]. H_2_O_2_ is a toxic lipid peroxidant and is further detoxified via conversion into water (H_2_O), which protects cells from the damaging effects of H_2_O_2_ accumulation from reactions catalysed by antioxidant enzymes, such as peroxidase (POD) and catalase (CAT), as well as the ascorbate-glutathione (AsA-GSH) cycle [6,7].

In the AsA-GSH cycle, ascorbate peroxidase (APX) catalyses the conversion of H_2_O_2_ to H_2_O with AsA as the electron donor and is accompanied by two molecules of monodehydroascorbate (MDHA) that are generated simultaneously [8]. Subsequently, monodehydroascorbate reductase (MDHAR) catalyses the conversion of MDHA to AsA, with NADPH as the electron donor [9]. MDHA may also rapidly disaggregate into dehydroascorbate (DHA), which is then reduced to AsA by catalysis with dehydroascorbate reductase (DHAR) with GSH as a substrate, thereby generating GSSG. Finally, glutathione reductase (GR) catalyses the reduction of oxidized GSSG into two molecules of GSH [10].

Plant growth regulators have been shown to contribute to the amelioration of negative impacts caused by drought stress [11]. Multiple investigations have indicated that a tertiary amine bioregulator known as 2-(3,4-dichlorophenoxy) triethylamine (DCPTA) regulates many aspects of plant development; for example, it promotes plant growth [12], enlarges chloroplast volume [13], enhances photosynthetic enzyme activity [14], accelerates CO_2_ fixation [15], and stimulates carotenoid biosynthesis [16]. However, few reports have focused on its effects in plants under stress conditions, and only a subset of these studies have focused on crops.

Maize (*Zea mays* L.), an important crop plant, is sensitive to drought stress, especially at seedling stage [17]. In maize, multigene families encode the AsA-GSH cycle enzymes. To date, seven APX genes (cytosolic APX1, cytosolic APX2, cytosolic APX4, peroxisomal APX3, mitochondrial APX5, mitochondrial APX6 and chloroplastic APX7), two GR genes (Cytosol GR1 and Cytosol GR2), three DHAR genes (Chloroplast DHAR1, Cytosol DHAR2 and Mitochondria DHAR3) and four MDHAR genes (Cytosol MDAR1, Cytosol MDAR2 and Cytosol MDAR3 and Mitochondrial MDAR4) have been identified [18]. Until now, it has remained unclear how the expression of the genes encoding the enzymes of the AsA-GSH cycle in maize responds to plant growth regulators.

In our research, we investigated the effects of DCPTA on the growth parameters of seedlings and the generation rate of O_2_·^−^; H_2_O_2_ and malondialdehyde (MDA) contents; electrolyte leakage (EL); non-enzymatic and enzymatic antioxidants; and the relative expression of genes encoding the isoenzymes of APX, MDHAR, DHAR and GR in the leaves of seedlings exposed to 15% polyethylene glycol (PEG)-6000 treatment to explore the modulation of the AsA-GSH cycle by DCPTA under drought stress.

## Materials and methods

### Plant material, growth conditions, design and sampling

This experiment was carried out in the growth chamber at Agricultural College, Northeast Agricultural University in Harbin, China. The DCPTA and maize inbred line Chang 7–2 were obtained from China Zhengzhou Zhengshi Chemical Company, Ltd. and Henan Academy of Agricultural Sciences, China, respectively.

After the germination (for 6 days), uniform seedlings were transferred to half-strength Hoagland’s nutrient solution (10 L) and maintained in opaque plastic containers (inner length, width, height: 50 cm, 30 cm, 18 cm; contained 60 seedlings) under controlled conditions: temperature, 25/18°C (day/night); light, 16/8 h (light/dark) period, 400 μmol m^-2^ s^-1^; relative humidity, 60~70%.

Maize seedlings (three-leaf stage) were subjected to the following four treatments: (1) control, untreated; (2) PEG, 15% (w/v) PEG-6000-simulated drought stress; (3) DCPTA, pre-treatment with DCPTA (15 mg l^–1^) added to the nutrient solution for 24 h under non-stress conditions (0% (w/v) PEG-6000); and (4) PEG+DCPTA, pre-treatment with DCPTA added to the nutrient solution for 24 h followed by exposure to 15% (w/v) PEG-6000-simulated drought stress. The doses of DCPTA (15 mg l^–1^) and PEG-6000 (15%) were selected according to our previous work [19]. The experiment was repeated five times. The nutrient solution was renewed and the pH was regulated to 6.50 (±0.05) every day. The nutrient solution was aerated daily at 7:00~9:00, 11:00~13:00 and 15:00~17:00.

Whole plants were sampled from each treatment on the 7^th^ day of drought stress for measurements of growth parameters. The second fully developed leaves of the seedlings were harvested on the 7^th^ day after drought stress for ultramicroscopic observations and histochemical staining of O_2_·^−^ and H_2_O_2_; at the 0, 1^st^, 3^rd^, 5^th^ and 7^th^ days after drought stress, portions of second fresh leaves were used to measure the electrolyte leakage (EL). The remaining leaves were immediately frozen in liquid nitrogen and stored at −80°C for later determination of other indicators.

### Measurements of relative growth rate (RGR)

After oven drying (105°C, 30 min), the shoots and roots were maintained at 80°C for 6 h; then, the shoot dry weight and root dry weight were measured. The RGR was determined as follows:

RGR = [ln (final dry weight)–ln (initial dry weight)]/(duration of treatment days) [20]. The date (S1 File) was used for analysis of variance.

### Measurements of ROS and MDA contents and EL

The formation rate of O_2_·^−^, H_2_O_2_ content, MDA content and EL were determined according to the methods of Elstner and Heupel (1976) [21], Jana and Choudhuri (1982) [22], Heath and Packer (1968) [23] and Lutts et al. (1995) [24], respectively. Histochemical staining of O_2_·^−^ and H_2_O_2_ were performed with nitroblue tetrazolium (NBT) and diaminobenzidine (DAB) solution, respectively, according to Chen et al. (2010) [25]. The date (S2 File) was used for analysis of variance.

### Measurements of antioxidant enzyme activities

To extract the antioxidant enzymes, frozen leaf samples (0.5 g) were homogenized using a chilled mortar and pestle with 8 ml of ice-cold 50 mM phosphate buffer (pH 7.8) and then immediately centrifuged (12 000×g for 20 min at 4°C). Phosphate buffer was added to the supernatant to a final volume of 5 ml, which was used for the antioxidant enzyme activity assays with a UV-visible spectrophotometer (Shimadzu, Japan).

The determination of protein concentration was performed according to Bradford (1976) using bovine serum albumin (BSA) as standard [26].

SOD activity (EC 1.15.1.1) was determined by measuring its ability to inhibit the photochemical reduction of NBT as described by Giannopolitis and Ries (1977) [27]. POD activity (EC 1.11.1.7) was measured according to the guaiacol method (Zheng and Huystee, 1992) [28]. CAT activity (EC 1.11.1.6) was measured as described by Aebi (1984) [29]. APX activity (EC 1.11.1.11) was measured following Nakano and Asada (1980) by monitoring the decrease in AsA absorbance at 290 nm [30]. GR activity (EC 1.6.4.2) was measured according to the decrease in absorbance at 340 nm caused by NADPH oxidation based on the method of Foyer and Halliwell (1976) [31]. MDHAR (EC 1.6.5.4) activity was measured according to the decrease in absorbance at 340 nm caused by NADH oxidation based on the method of Pyngrope et al. (2013) [32]. DHAR activity (EC 1.8.5.1) was measured according to the increase in absorbance at 265 nm caused by AsA formation based on the method of Nakano and Asada (1980) [30]. The date (S3 File) was used for analysis of variance.

### Measurements of AsA/DHA and GSH/GSSG in leaves

Frozen leaf samples (0.5 g) were ground in 5 ml of 5% (v/v) ice-cold phosphoric acid and then centrifuged (13000 × g, 20 min, 4°C). The supernatant was then used to determine the contents of AsA, total AsA, DHA, GSSG, total GSH, and GSH. The levels of AsA and DHA were determined as described by Hodges et al. (1997) [33]. The GSSG and total GSH contents were measured as described by Griffith (1980)[34]. The date (S4 File) was used for analysis of variance.

### RNA isolation and real-time RT-PCR

Total RNA was isolated from the maize leaves using TRIzol reagent (Invitrogen, Carlsbad, CA, USA). Specific primers for each gene were designed from the 3’ ends of the gene sequences (Table 1). The synthesis of cDNA and real-time PCR were performed as previously described of Liu et al. (2012) [18]. The 2^−ΔΔCt^ method was used to calculate the relative transcript levels. The date (S5 File) was used for analysis of variance.

**Table 1:** Primer sequences for real-time RT-PCR.

Gene	Forward sequence	Reverse sequence
**ZmAPX1.1**	5’-TGATGCCACTAAGGGTTCT-3’	5’-ATCACTCCAGGATAGGGTCT-3’
**ZmAPX1.2**	5’-TTCAGCTCCCAAGTGACAAA-3’	5’-TCTAGGCAAACGGAAAATGG-3’
**ZmAPX2**	5’-CTCAGGCAGGTTTTCTCCAC-3’	5’-GGATCAGAGAGGAGGGCTTT-3’
**ZmAPX3**	5’-CCAGATCTGCGAATAAACACAA-3’	5’-AAATACATGTGCACAGAACTGAAA-3’
**ZmAPX4**	5’-GAGGTCTGGATTCGATGGTG-3’	5’-CTGATTTGGATGGTGCTGTG-3’
**ZmAPX5**	5’-GATGCTGTGCTGTTTGAGGA-3’	5’-ACAGGGCACGCTAAGAAAAA-3’
**ZmAPX6**	5’-GCAGGGATTCTCTTTGGATG-3’	5’-GCCACTGTGTCGGTTCTTTT-3’
**ZmAPX7**	5’-TGCTAAGCTGAGCGATCTTG-3’	5’-TACTCCGCCCTGATCTTTTG-3’
**ZmGR1**	5’-CGGTGCAATAGTGGTTGATG-3’	5’-CCTATTGGTGGTTGGGAGAA-3’
**ZmGR2**	5’-CGATATTGCGGTTAAATGTG-3’	5’-AAGTTCGTCTTTGGCTTGGA-3’
**ZmDHAR1**	5’-CATCAAGACTAAGCCCACCAA-3’	5’-TAGAAACATGGCCACCACAA-3’
**ZmDHAR2**	5’-CAATGTCCATGCCTACACCA-3’	5’-CAGGTAGCACCAAAGCACAA-3’
**ZmDHAR3**	5’-CGAGGAAAAATGGATTGGTG-3’	5’-TGTTCCATCGCTTGGATCTT-3’
**ZmMDAR1**	5’-TACTCCCGATCATTCGACCT-3’	5’-GGCAATGACCTTGTTCTCGT-3’
**ZmMDAR2**	5’-TCAAGGAGCAGAATCCAACA-3’	5’-GCCCTATGTAACCACCTCCA-3’
**ZmMDAR3**	5’-CAGCTCTGTGTATGCCGTTG-3’	5’-ATCGATGTCCCTCGTCTTTG-3’
**ZmMDAR4**	5’-GTGCAAAGAAGGTGGTGGTT-3’	5’-TTCTTAGCAAGCGAGGGTGT-3’
**ZmTUB**	5’-GCTATCCTGTGATCTGCCCTGA-3’	5’-CGCCAAACTTAATAACCCAGTA-3’

### Statistical analysis

The experimental data were expressed as the means and standard deviations. Statistical analyses were performed using SPSS 15.0 and Excel 2007, and the means were determined using Fisher’s LSD test at a significance level of P<0.05.

## Results

### Effects of PEG and/or DCPTA application on the relative growth rate (RGR) of shoots and roots

**Fig 1:**
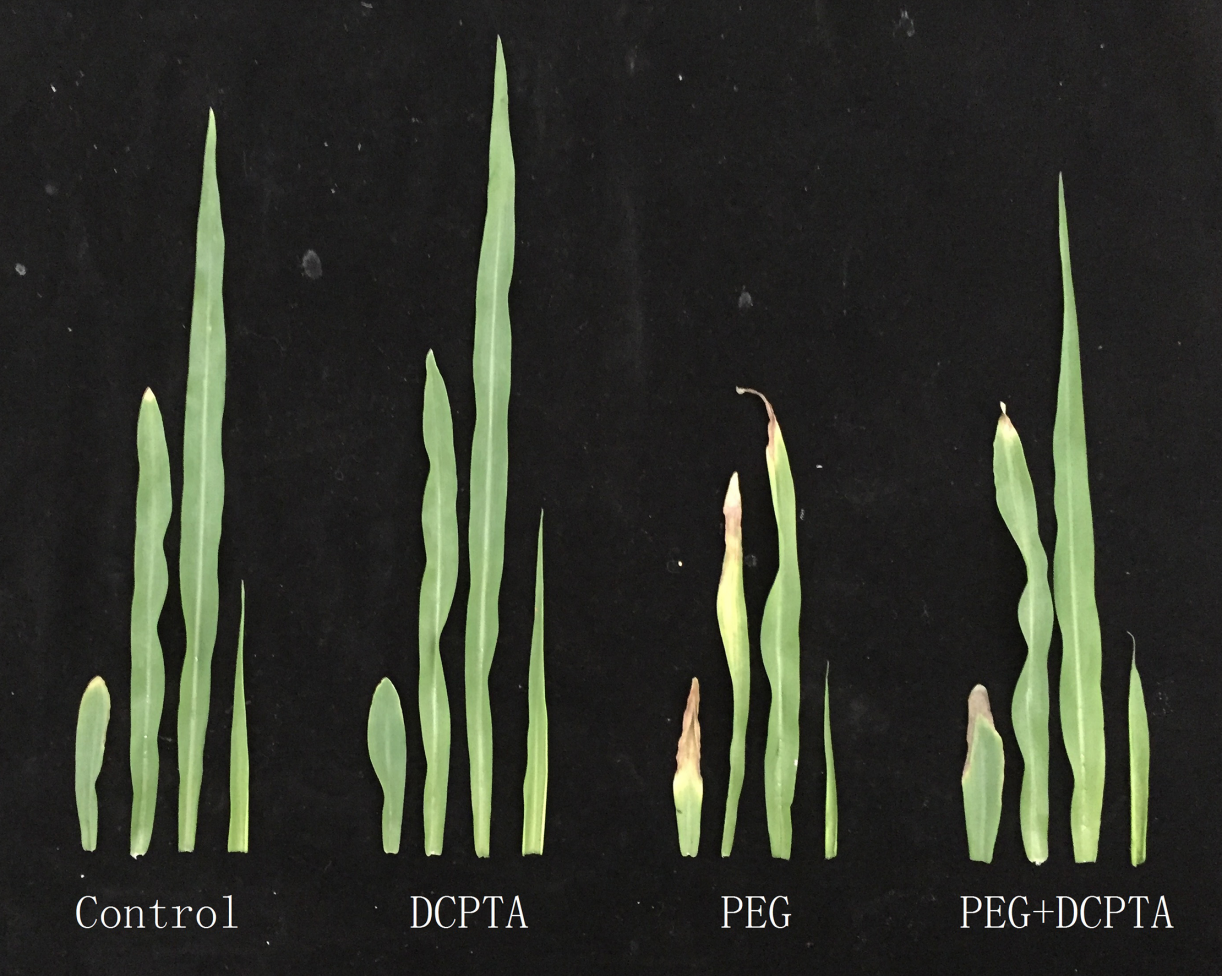
Leaf phenotype characteristics of the maize seedlings after 7 days of treatment with DCPTA and/or PEG-6000.

**Fig 2:**
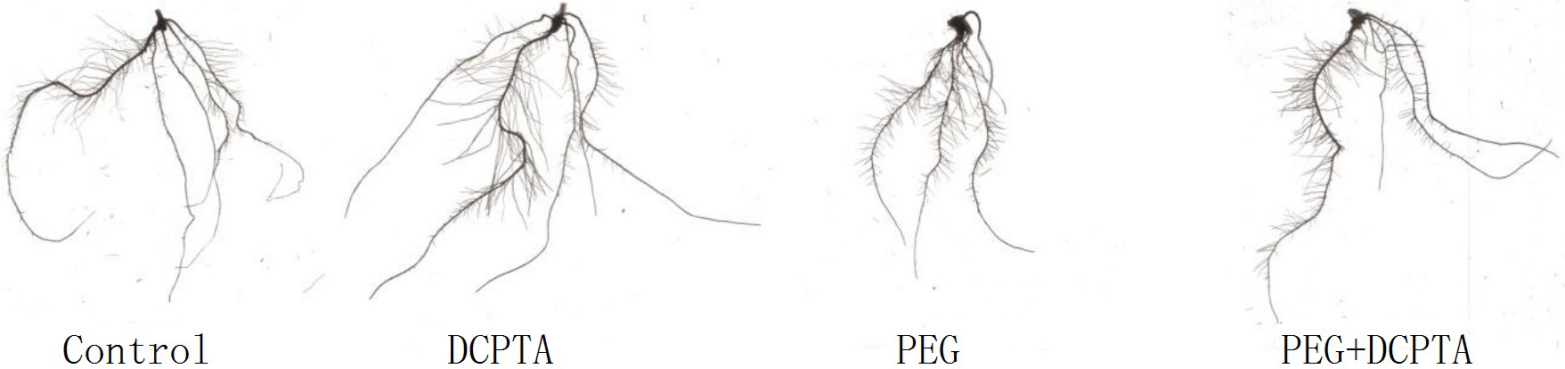
Root phenotype characteristics of the maize seedlings after 7 days of treatment with DCPTA and/or PEG-6000.

After 7 days of exposure to 15% PEG-6000-simulated drought, the plants exhibited growth inhibition; however, exogenous DCPTA partially alleviated this growth inhibition. DCPTA also positively affected the morphology of maize seedlings under non-stress conditions (Table 2, Figs 1 and 2). Compared with that of the control, the RGRs of the shoots and roots decreased by 35.26% and 27.99% in response to 15% PEG-6000 treatment and by 21.60% and 16.28% in response to PEG + DCPTA treatment, but increased by 9.54% and 8.22% in response to DCPTA treatment, respectively.

**Table 2:** Effects of exogenous DCPTA application on the relative growth rate (RGR) of the shoots and roots of maize seedlings exposed to PEG-induced drought stress for 7 days.

Treatment	Shoot RGR (mg DW day^-1^)	Root RGR (mg DW day^-1^)
**Control**	79.64±4.13 b	78.88± 3.23 b
**15 mg L**^**–1**^ **DCPTA**	87.24±2.87 a	85.36± 1.58 a
**15% PEG-6000**	51.56±5.55 d	56.80± 2.61 d
**15% PEG-6000 + 15 mg L**^**–1**^ **DCPTA**	62.44±2.65 c	66.04± 2.46 c

The values represent the mean±SE (n = 5). Values with the same letters in the columns are not significantly different at P<0.05 (LSD test).

### Effects of PEG and/or DCPTA application on the generation rate of O_2_·^−^, H_2_O_2_ content, MDA content and EL

**Fig 3:**
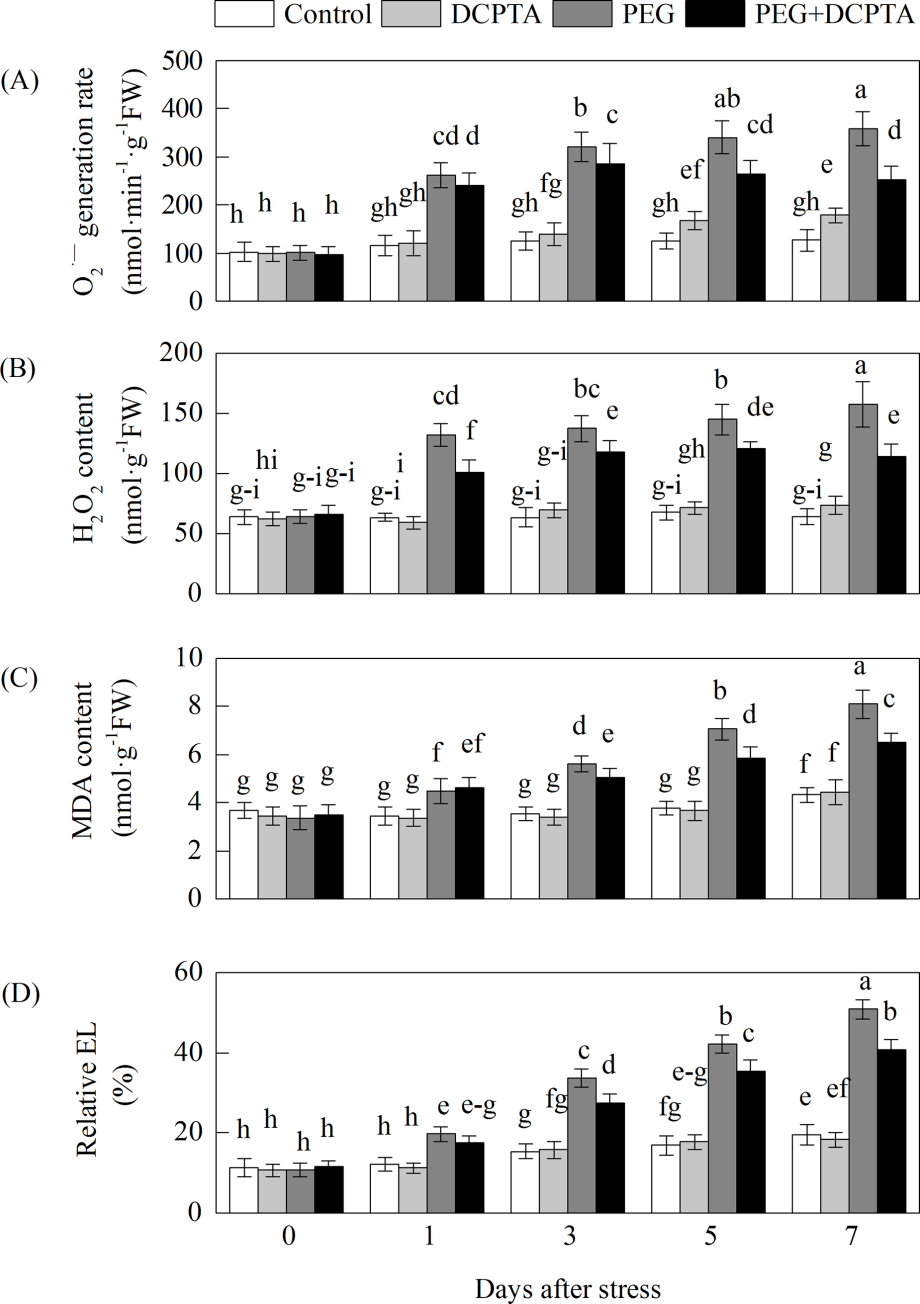
**Effects of exogenous DCPTA application on the O**_**2**_**·**^**−**^ **generation rate (A), H**_**2**_**O**_**2**_ **content (B), MDA content (C) and EL (D) in leaves of maize seedlings exposed to PEG-induced drought stress for 7 days.** The data represent the means of independent measurements for five replicates, and the standard deviations are indicated by vertical error bars. Values with the same letters on the bars are not significantly different at P<0.05.

**Fig 4:**
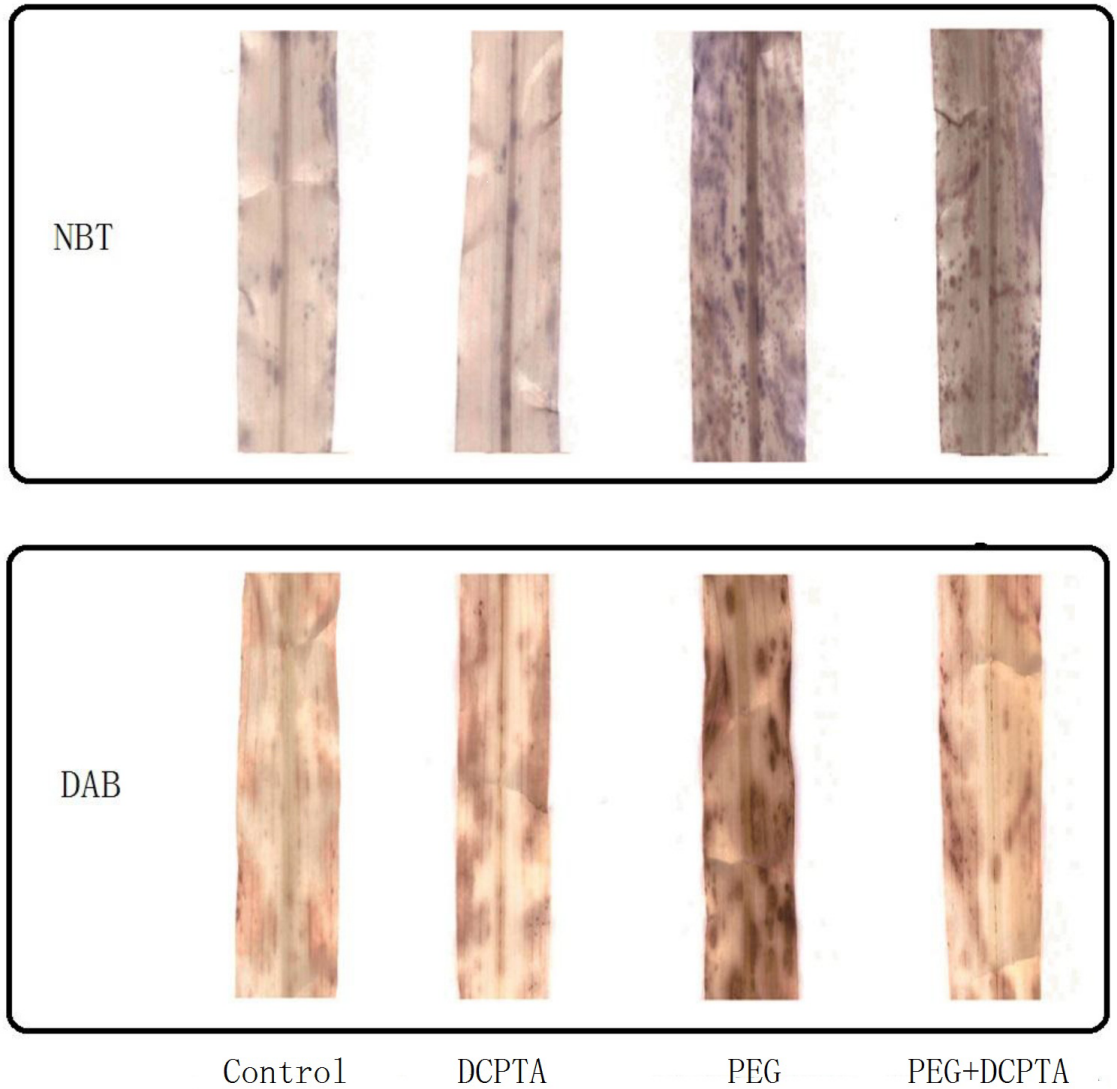
Histochemical localization of O_2_·^−^ (NBT) and H_2_O_2_ (DAB) in the leaves of maize seedlings exposed to PEG-induced drought stress for 7 days.

Drought stress increased the generation rate of O_2_·^−^ by 125.90%, 156.75%, 171.96% and 184.84% and the H_2_O_2_ content by 107.69%, 116.44%, 114.16%, and 144.28% on the 1^st^, 3^rd^, 5^th^ and 7^th^ days, respectively, relative to the corresponding control values (Fig 3). Compared with PEG treatment alone, DCPTA treatment decreased the generation rate of O_2_·^−^ by 8.09%, 11.10%, 22.30% and 29.83% and the H_2_O_2_ content by 22.98%, 14.62%, 16.80% and 27.55% on the 1^st^, 3^rd^, 5^th^ and 7^th^ days, respectively. The histochemical staining of O_2_·^−^ and H_2_O_2_ in the maize leaves showed similar results. A reduced density of blue and brown spots indicated lesser accumulation of O_2_·^−^ and H_2_O_2_ in the DCPTA-treated leaves than in the non-treated leaves under drought stress (Fig 4). In the PEG treatment, MDA content and EL increased regardless of whether DCPTA was applied on the 1^st^ day; however, the seedlings treated with DCPTA had significantly lower MDA contents and EL by the 3^rd^, 5^th^ and 7^th^ days of drought stress than did the non-DCPTA-treated ones. By the 7^th^ day, compared with the control values, MDA content and EL had increased by 86.85% and 160.79%, respectively, in the PEG treatment and by 50.66% and 108.40%, respectively, in the PEG+DCPTA treatment. DCPTA application had no significant effect on EL or the contents of H_2_O_2_ and MDA, but it slightly increased the generation rate of O_2_·^−^ under non-stress conditions.

### Effects of PEG-6000 and/or DCPTA application on antioxidant enzymes activities

**Fig 5:**
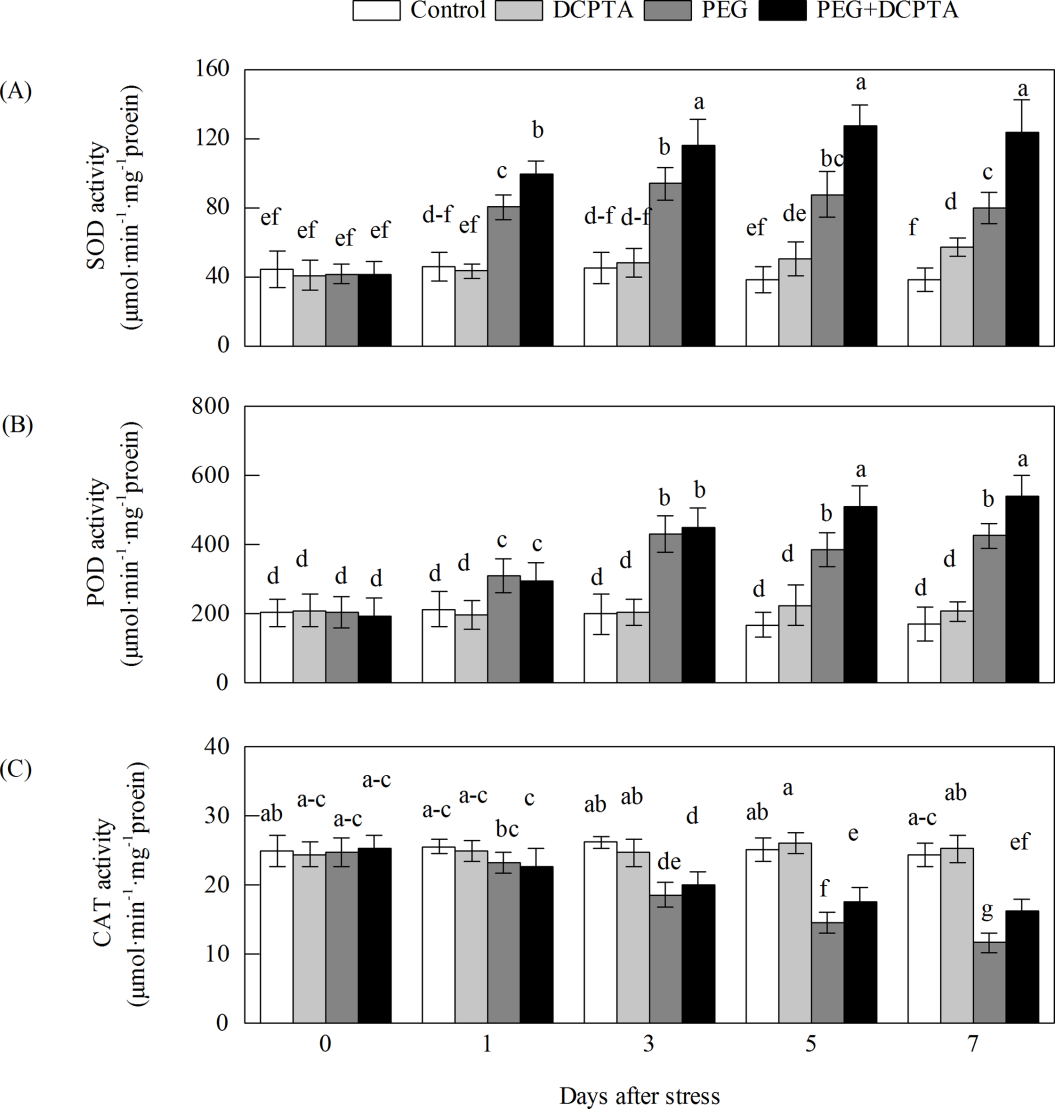
**Effects of exogenous DCPTA application on the activities of SOD (A), POD (B), and CAT (C) in the leaves of maize seedlings exposed to PEG-induced drought stress for 7 days.** The data represent the means of independent measurements for five replicates, and the standard deviations are indicated by the vertical error bars. Values with the same letters on the bars are not significantly different at P<0.05.

**Fig 6:**
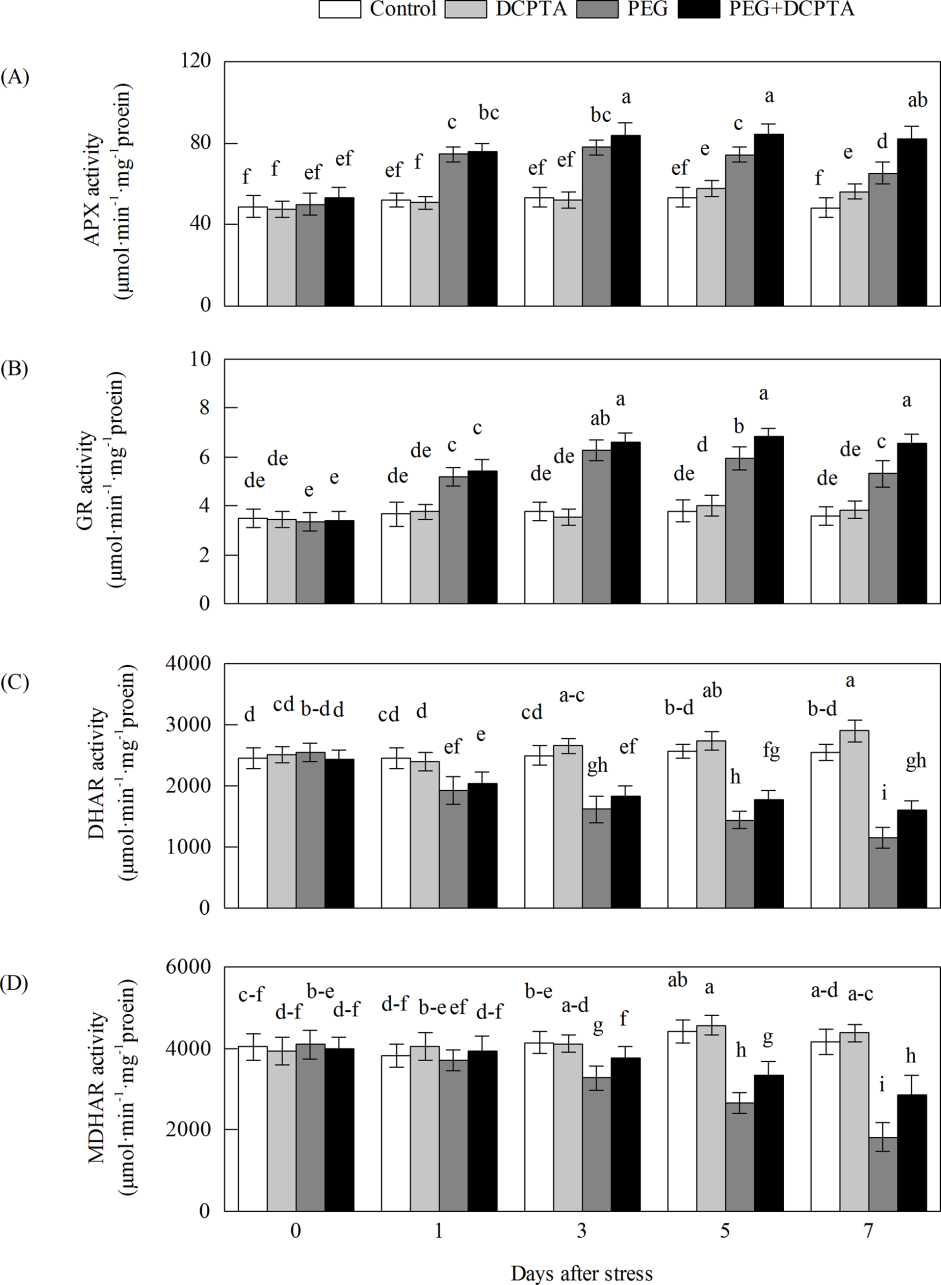
**Effects of exogenous DCPTA application on the activities of APX (A), GR (B), DHAR (C), and MDHAR (D) in the leaves of maize seedlings exposed to PEG-induced drought stress for 7 days.** The data represent the means of independent measurements for five replicates, and the standard deviations are indicated by vertical error bars. Values with the same letters on the bars are not significantly different at P<0.05.

From 0 to the 1^st^ day under drought stress alone, the SOD, POD, APX and GR activities increased rapidly and peaked on the 3^rd^ day; thereafter, the SOD, APX and GR activities decreased, whereas POD activity remained relatively stable (Figs 5 and 6). The activities of SOD, POD, APX and GR in plants pre-treated with DCPTA were increased compared with those in plants under drought stress alone. The activities of SOD from the 1^st^ day, APX from the 3^rd^ day, and POD and GR from the 5^th^ day showed significant differences between the PEG and PEG+DCPTA treatments. At the 7^th^ day, the SOD, POD, APX and GR activities were increased by 107.45%, 148.76%, 35.46%, and 48.17%, respectively, under the PEG treatment and by 221.90%, 216.41%, 70.12%, and 71.13%, respectively, under the PEG+DCPTA treatment relative to the control values. The CAT, MDHAR and DHAR activities gradually decreased as the drought stress continued. However, the DCPTA applications depressed the inhibition of the CAT, MDHAR and DHAR activities. At the 7^th^ day, the CAT, MDHAR and DHAR activities were decreased by 52.20%, 56.12%, and 54.61%, respectively, under the PEG treatment and by 33.59%, 31.16%, and 36.73%, respectively, under the PEG+DCPTA treatment relative to the control values. Under non-stress conditions, DCPTA increased the SOD, APX and DHAR activities but had no significant effect on the activity of POD, CAT, GR or MDHAR. The SOD, APX, and DHAR activities were increased by 49.02%, 16.61% and 13.71%, respectively, under the DCPTA treatment relative to the control values.

### Effects of PEG-6000 and/or DCPTA application on non-enzymatic antioxidants

**Fig 7:**
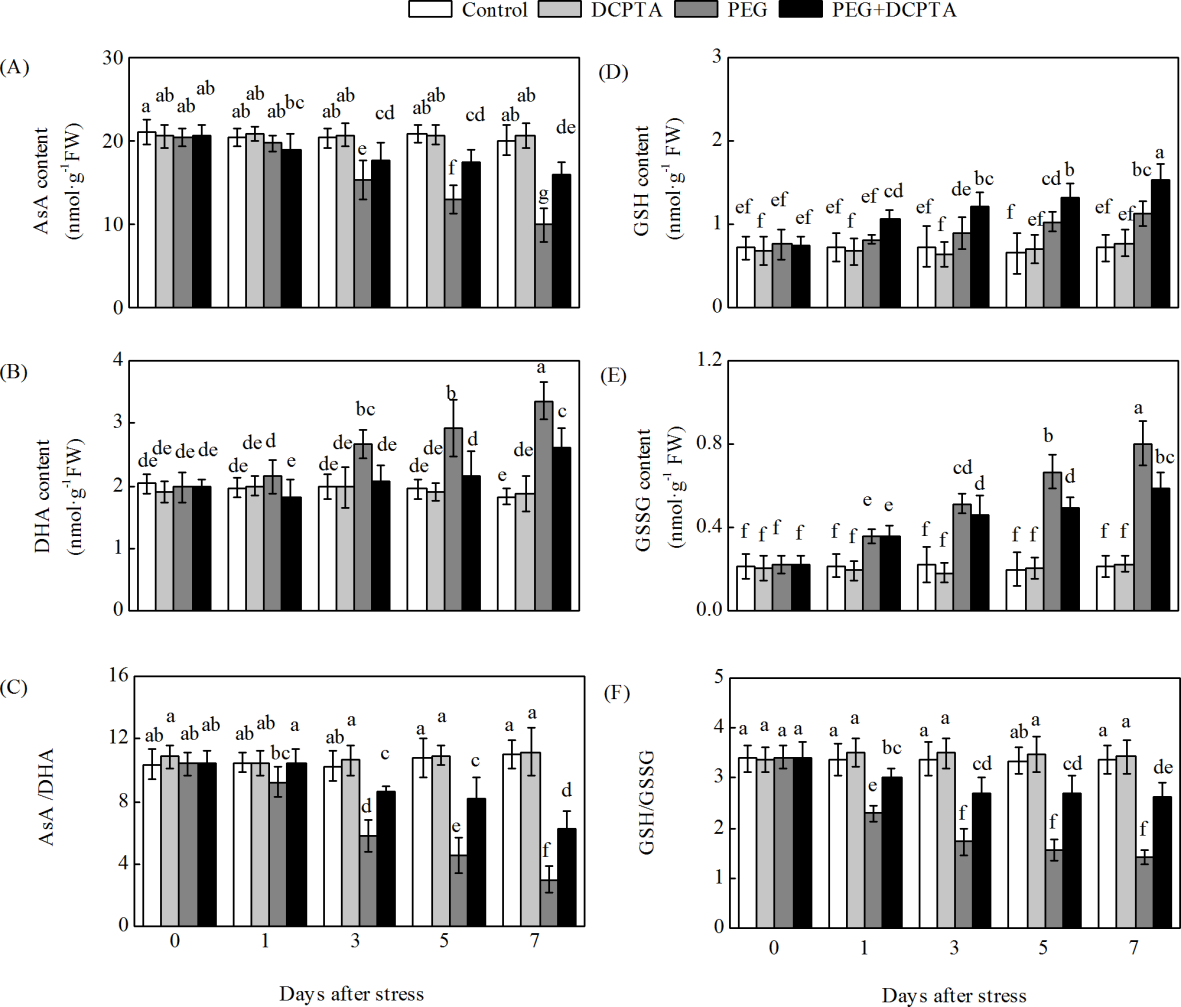
**Effects of exogenous DCPTA application on the contents of AsA (A) and DHA (B), the AsA/DHA ratio (C), reduced GSH (D), oxidized GSSG (E), and the GSH/GSSG ratio (F) in leaves of maize seedlings exposed to PEG-induced drought stress for 7 days.** The data represent the means of independent measurements from five replicates, and the standard deviations are indicated by vertical error bars. Values with the same letters on the bars are not significantly different at P<0.05.

After the 3^rd^ day, the drought stress significantly decreased the AsA content and the AsA/DHA ratio and increased the DHA content relative to the control values (Fig 7). Under drought stress, increases relative to the control treatment were observed in the GSH and GSSG contents, whereas a decrease was observed in the GSH/GSSG ratio. DCPTA application increased the AsA content and the AsA/DHA ratio and decreased the DHA content in the drought-stressed maize seedlings relative to the values observed in the seedlings exposed to drought stress only. DCPTA application also increased the GSH/GSSG ratio by increasing the GSH content and reducing the GSSG content in the PEG-affected seedlings. The AsA, DHA, GSH, and GSSG contents and the AsA/GSH and GSH/GSSG ratios were not significantly different between the non-treated and DCPTA-treated plants under non-stress conditions.

### Effects of PEG-6000 and/or DCPTA applications on the expression of genes encoding the AsA-GSH cycle enzymes

**Fig 8:**
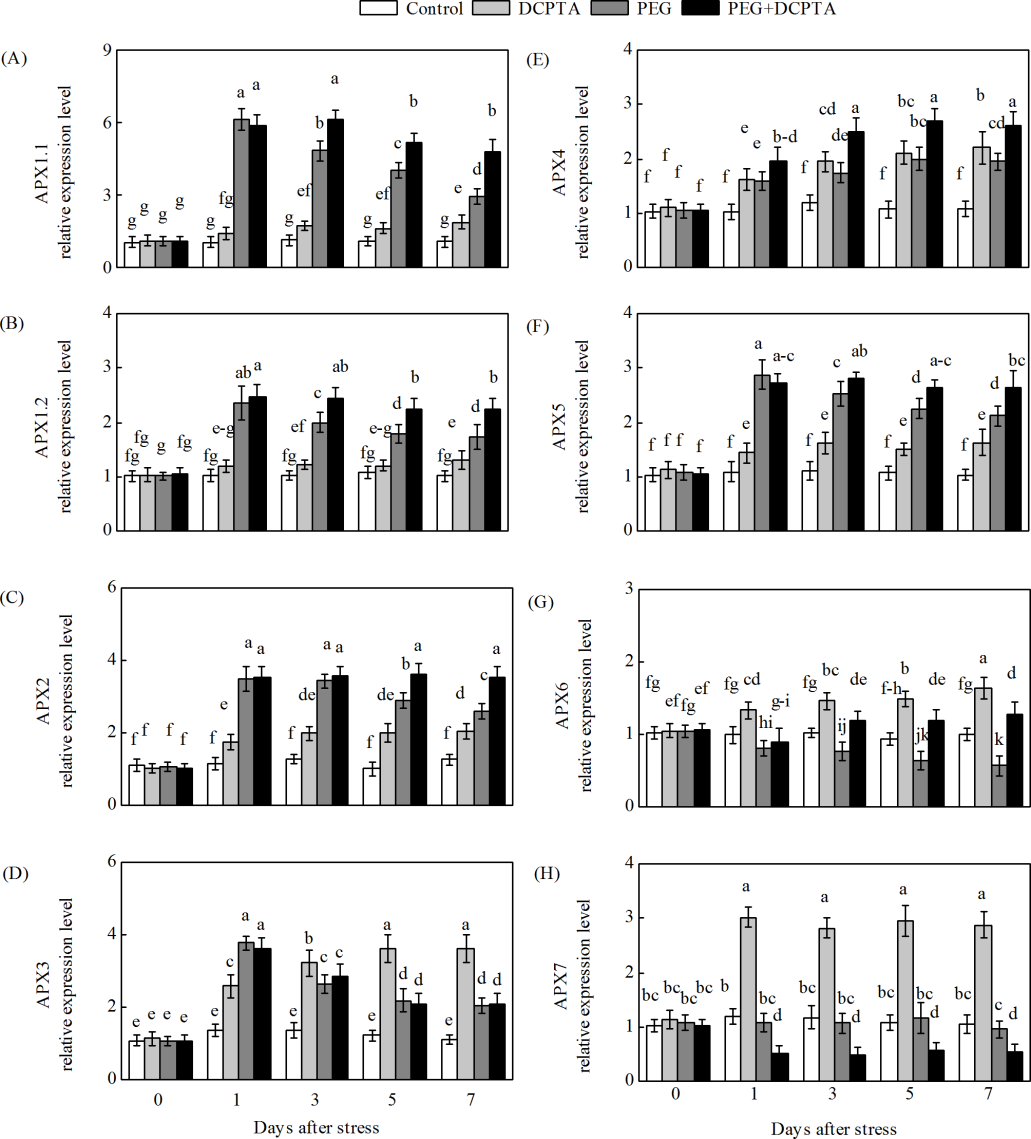
**Effects of exogenous DCPTA application on relative expression level of APX1.1 (A), APX1.2 (B), APX2 (C), APX3 (D), APX4 (E), APX5 (F), APX6 (G) and APX7 (H) in the leaves of maize seedlings exposed to PEG-induced drought stress for 7 days.** The data represent the means of independent measurements from five replicates, and the standard deviations are indicated by vertical error bars. Values with the same letters on the bars are not significantly different at P<0.05.

**Fig 9:**
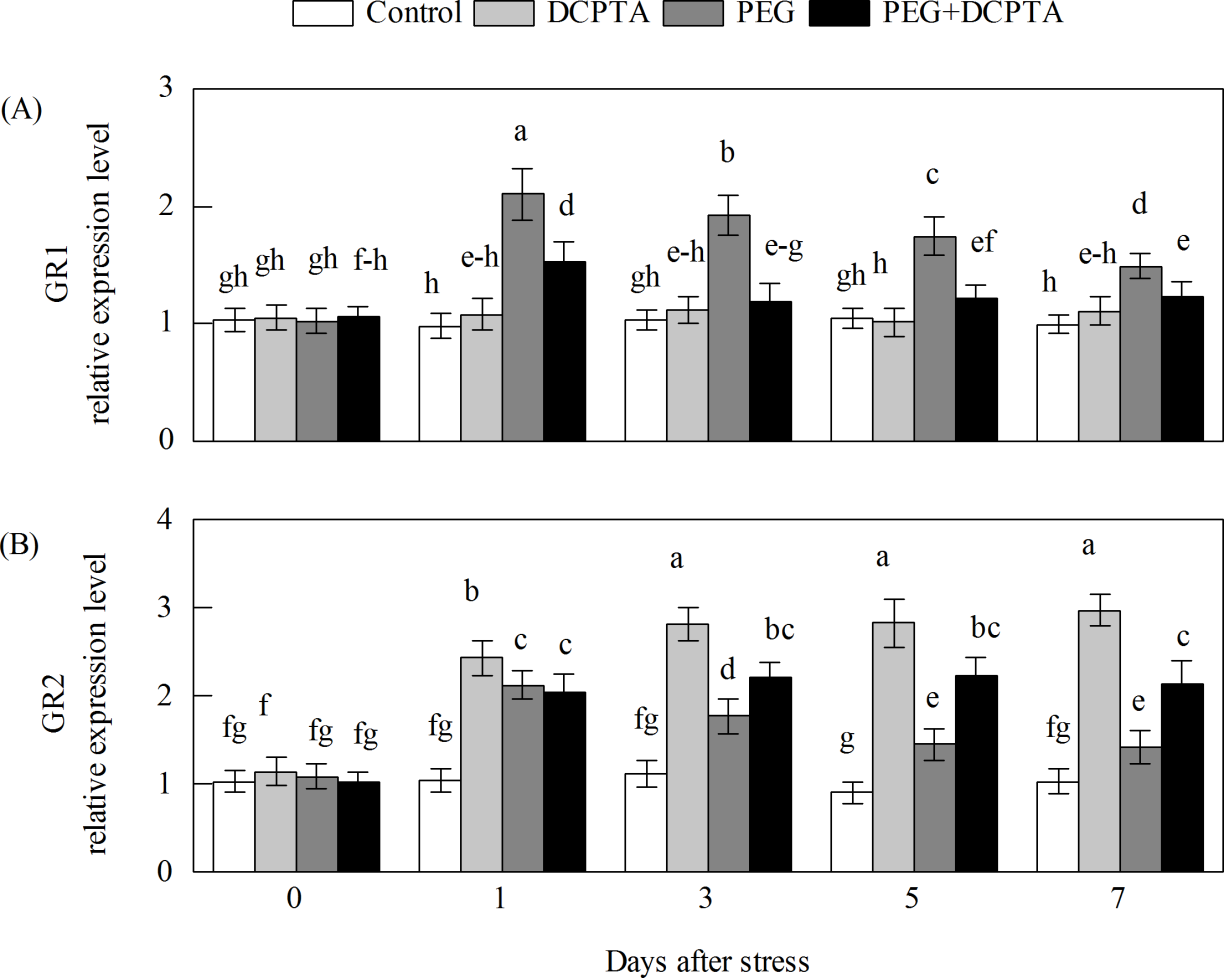
**Effects of exogenous DCPTA application on the relative expression level of GR1 (A) and GR2 (B) in the leaves of maize seedlings exposed to PEG-induced drought stress for 7 days.** The data represent the means of independent measurements for five replicates, and the standard deviations are indicated by vertical error bars. Values with the same letters on the bars are not significantly different at P<0.05.

**Fig 10:**
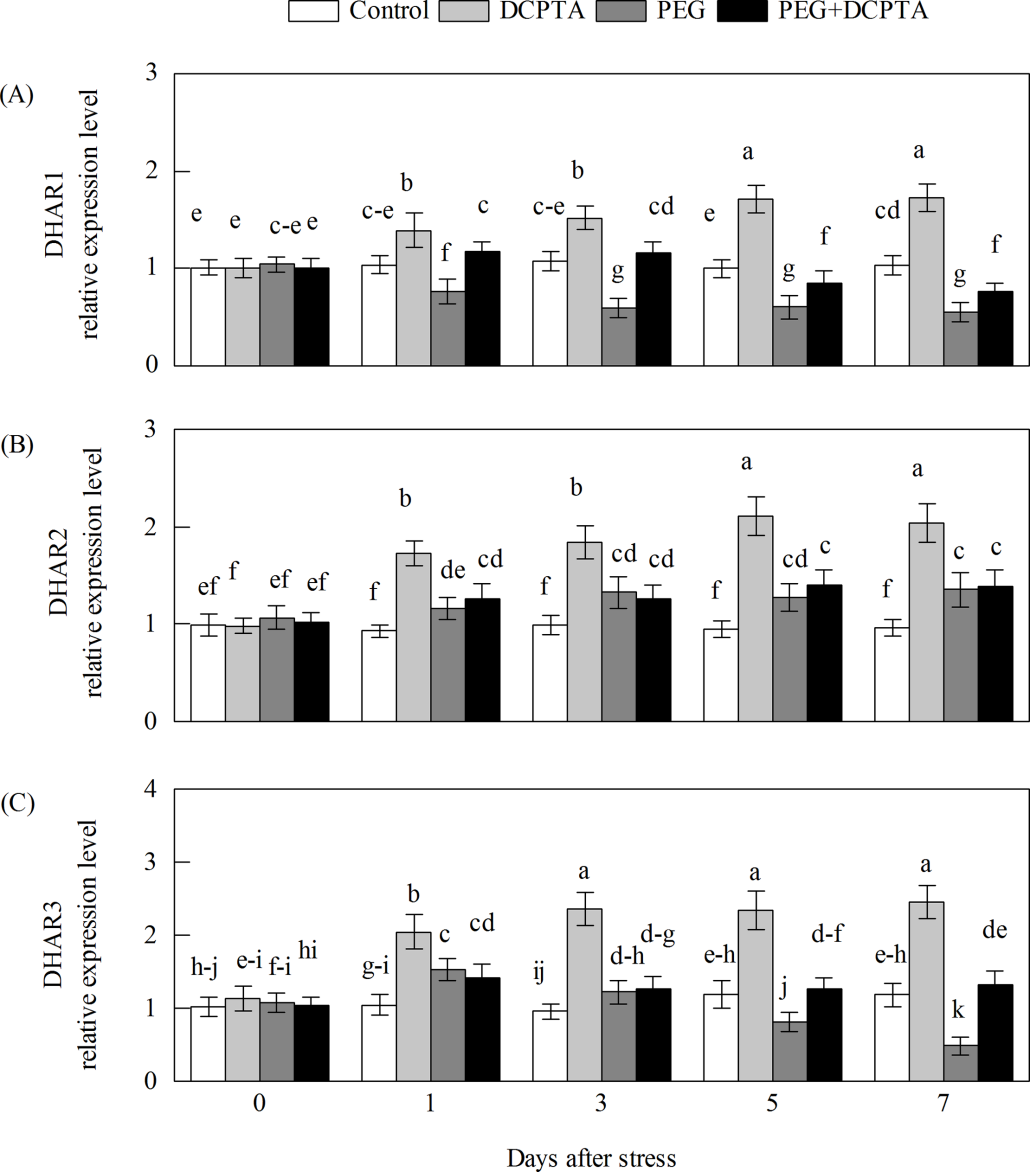
**Effects of exogenous DCPTA application on the relative expression level of DHAR1 (A), DHAR2 (B) and DHAR3 (C) in the leaves of maize seedlings exposed to PEG-induced drought stress for 7 days.** The data represent the means of independent measurements for five replicates, and the standard deviations are indicated by vertical error bars. Values with the same letters on the bars are not significantly different at P<0.05.

**Fig 11:**
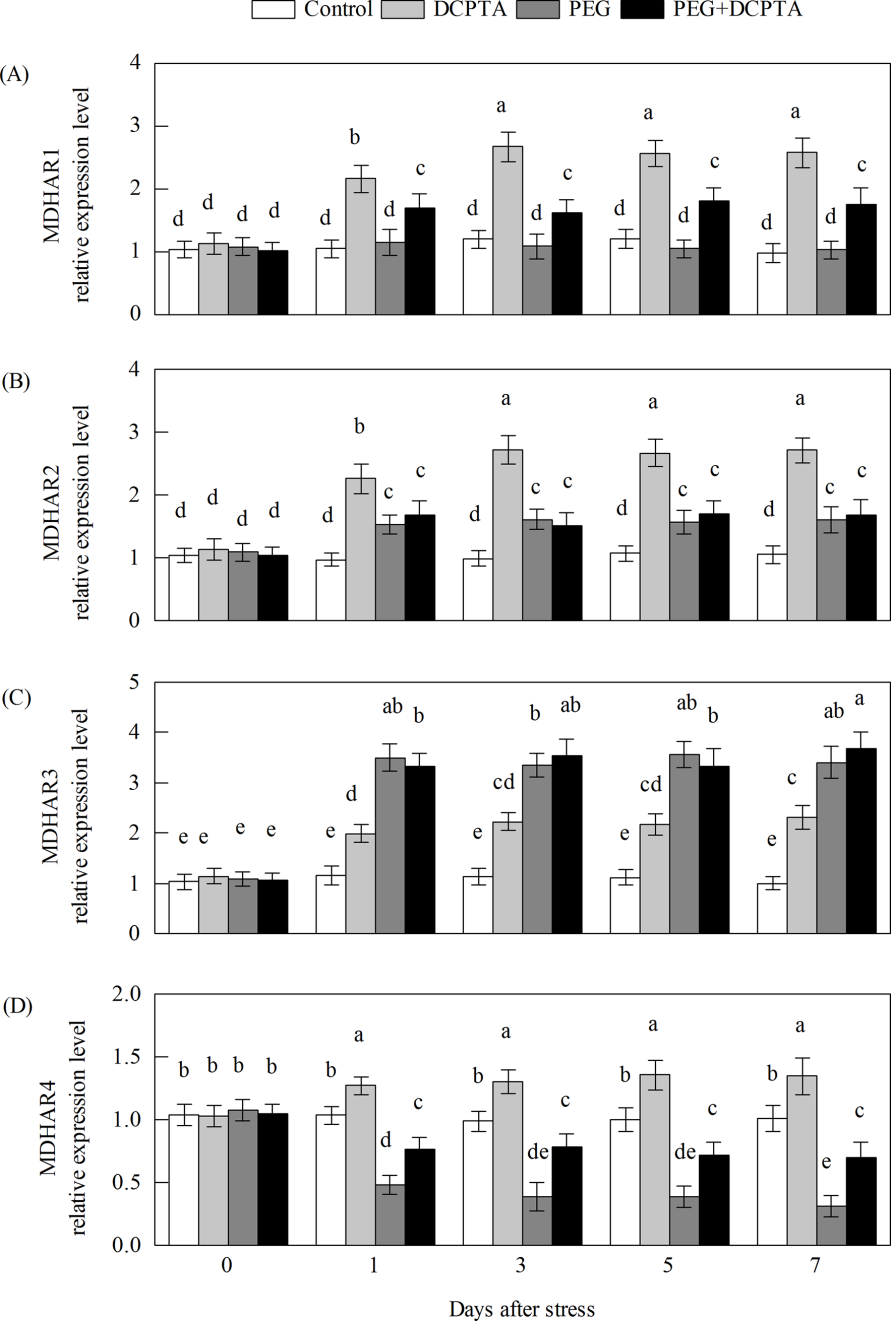
**Effects of exogenous DCPTA application on the relative expression level of MDHAR1 (A), MDHAR2 (B), MDHAR3 (C) and MDHAR4 (D) in the leaves of maize seedlings exposed to PEG-induced drought stress for 7 days.** The data represent the means of independent measurements for five replicates, and the standard deviations are indicated by the vertical error bars. Values with the same letters on the bars are not significantly different at P<0.05.

The transcripts of all of the maize genes except GR1 in the DCPTA-treated seedlings showed a steady-state increase under non-stress conditions (Figs 8–11). The transcript levels of APX1.1, APX1.2, APX2, APX3, APX4, APX5, GR1, GR2 and DHAR3 first increased and then decreased under the 15% PEG-6000 drought stress treatment, whereas DCPTA application increased the transcript levels of APX1.1, APX1.2, APX2, APX4, APX5, GR2 and DHAR3, had no significant effects on the transcript level of APX3 and decreased the transcript level of GR1. The transcript levels of DHAR2, MDHAR2 and MDHAR3 initially increased and then remained unchanged under the 15% PEG-6000 drought stress treatment, and DCPTA application had no significant effect on the transcript level of DHAR2, MDHAR2 or MDHAR3. The transcript levels of APX6, DHAR1, and MDHAR4 were decreased under the 15% PEG-6000 drought stress treatment and increased by DCPTA application. The 15% PEG-6000 drought stress treatment had no significant effect on the APX7 and MDHAR1 transcripts, whereas the APX7 transcripts were decreased by DCPTA application and the MDHAR1 transcripts were increased by DCPTA application.

## Discussion

Growth promotion following the application of the tertiary amine bioregulator DCPTA has been observed in many plant species, including soybean, radish, cotton, sugar beet, and tomato [14]. In this work, DCPTA application had a similar effect on maize seedlings under non-stress conditions; this effect might have been caused by the promotion of CO_2_ fixation, as previously observed in cotton [15]. PEG, a polyether compound, is inert, non-ionic and cell impermeable, and it has been widely applied to simulate drought stress [35]. Moreover, in the present study, DCPTA-pre-treated seedlings were more tolerant of drought stress than were the non-treated seedlings, as evidenced by the increased RGR of shoots and roots (Table 2, Figs 1 and 2).

Excessive levels of ROS induced by stress lead to the oxidation of cellular components, which ultimately results in plant growth inhibition [36]. In this study, drought stress significantly increased MDA content and EL, which may be due to the accumulation of O_2_·^−^ and H_2_O_2_ in the leaves of maize seedling (Fig 3), resulting in injury to the biological membrane system (had observed in prior results reported by our research group), leading to the interruption of metabolism [19,37,38]. Therefore, the increased generation rate of O_2_·^−^ and the excess amount of H_2_O_2_ might partly explain the growth inhibition of the seedlings in the 15% PEG-6000-induced drought stress treatment in this study. The addition of DCPTA to the 15% PEG-6000 treated maize seedlings reduced the generation rate of O_2_·^−^ and the magnitude of H_2_O_2_, and the in vivo detection of O_2_·^−^ and H_2_O_2_ via NBT and DAB staining further illustrated the low accumulation of O_2_·^−^ and H_2_O_2_ (as indicated by dark blue and deep brown spots) in the leaves of plants in the PEG+DCPTA treatment (Fig 4). Moreover, DCPTA application decreased the levels of membrane lipid peroxidation and membrane leakiness as expressed by the lower MDA content and lower EL (Fig 3). These results suggest that DCPTA protected the maize seedlings from oxidative damage by reducing the accumulation of O_2_·^−^ and H_2_O_2_. These changes maintained the membrane integrity and stability, which are the major components of resistance in plants, thereby mitigating the growth inhibition induced by drought stress [39]. Under non-stress conditions, the generation rate of O_2_·^−^ and the accumulation of H_2_O_2_ were not decreased in the DCPTA-treated seedlings; interestingly, the generation rate of O_2_·^−^ was significantly higher in these seedlings than in the controls despite the increased SOD activity (Fig 5). One possible reason for this finding is that the application of DCPTA promoted photosynthesis and thus ROS accumulation. Moreover, the increased plant protection from oxidative damage in response to DCPTA application might be attributed to the suppression of ROS generation and/or the enhanced antioxidant defence systems under drought stress [40].

In this study, SOD activity increased rapidly and then decreased slowly in the seedling leaves over the drought stress period (Fig 3), which might have been part of the plants' adaptive response to stress [36]. Because SOD is a substrate-inducible enzyme, the enhanced SOD activity of the seedlings under drought stress may have been caused by the increased accumulation of O_2_·^−^ as a substrate [41]. However, the simultaneous increase in the O_2_·^−^ generation rate indicated that the enhanced SOD activity did not provide sufficient protection against oxidative stress (Figs 3 and 5). The further enhanced SOD activity of maize seedlings under the PEG+DCPTA treatment may have increased the capacity to scavenge O_2_·^−^, which is consistent with the significantly decreased level of O_2_·^−^ accumulation (Figs 3 and 4). In our study, POD activity increased and CAT activity decreased upon exposure to continued drought stress (Fig 5), which corroborates the findings of other researchers [42]. The decreased CAT activity may have been related to inactivation due to the accumulated H_2_O_2_ induced by drought stress [43]. Here, POD activity was further elevated and CAT activity partially recovered due to DCPTA application, which indicates that DCPTA can maintain a high efficiency of ROS quenching to limit H_2_O_2_ accumulation under drought stress [44]. The elevated CAT activity may have been caused by the lower H_2_O_2_ accumulation in the DCPTA pre-treated seedlings under drought stress.

In plants, ascorbate (AsA) and glutathione (GSH) are major non-enzymatic antioxidants. Their concentrations and the statuses of oxidation and reduction are tightly associated with plant stress tolerance [45,46]. Higher contents of AsA and GSH correspond to reduced ROS-associated injuries in plants [47]. The AsA-GSH cycle is the key defence mechanism for regulating the oxidative and reductive environments via the modulation of the interconversion of AsA/DHA and GSH/GSSG in the foliar tissues of higher plants [48].

AsA is potent water-soluble ROS scavenger of cells, converting H_2_O_2_ to H_2_O by APX [8]. A high level of AsA is essential to maintain the antioxidant capacity that protects plants from oxidative stresses [49]. The enzymes MDHAR and DHAR are the key components in the regeneration of AsA from DHA through the electron donors NADPH and GSH [7]. Alterations in the activities of MDHAR and DHAR profoundly influence the intensity of AsA recycling [50,51]. In this work, the disturbed AsA synthesis induced by drought stress could partly be attributed to the decreases in MDHAR and DHAR activities (Fig 6). It has been reported that MDHAR overexpression can increase the resistance to oxidative stress [52,53]. In addition, previous studies have shown that the overexpression of DHAR genes is beneficial in elevating tolerance towards heavy metal exposure, drought stress and salt stress because it allows the maintenance of high AsA levels [18,54,55,56]. In the present work, the DCPTA pre-treatment resulted in significant increases in MDHAR and DHAR activities and MDHAR1 and MDHAR4 transcript levels, which may be primary causes of the maintenance of lower levels of DHA and higher levels of AsA as well as higher AsA/DHA ratios (compared to drought stress alone) [57,58].

Increases in APX activity and transcript expression in the foliar tissues of higher plants exposed to osmotic treatment have been shown in previous studies [18,59]. In this study, the transcripts of different ZmAPX genes showed varying responses in the maize seedlings treated with 15% PEG-6000 (Fig 8). The transcripts of ZmAPX 1.1, ZmAPX 1.2, ZmAPX 2, ZmAPX 3, ZmAPX 4 and ZmAPX 5 increased in response to drought stress, with ZmAPX1.1 showing the strongest response, whereas the transcripts of APX 6 decreased and those of APX 7 remained unaltered. These results suggest that compared with the other ZmAPXs, ZmAPX1.1 likely plays a more important role in maize exposed to drought stress. APX activity is directly dependent on AsA availability, and the enhanced APX activity and up-regulated transcript expression levels of the APXs (except APX 7) in the DCPTA-treated seedlings during drought stress might have been dependent on the regeneration and/or biosynthesis of AsA, which is regulated by MDHAR and DHAR (Figs 6 and 8) [60].

GR, the rate-limiting enzyme, is responsible for catalysing the conversion of GSSG to GSH via NADPH in the AsA-GSH cycle. This process favours AsA reduction and protects cells from being damaged by ROS by maintaining a favourable GSH/GSSG ratio for cellular redox regulation [61]. Previous studies have indicated that GR activity is regulated by stress and that GR overexpression can enhance plant stress resistance [62,63]. Under drought stress, the GSH level increased slightly, but the GSSG level increased sharply, which resulted in a decreased GSH/GSSG ratio compared to the control ratio. This result may be attributed to the GR and DHAR changes induced by drought stress (Figs 6, 7D and 9) [42,64]. Furthermore, DCPTA application further increased the GR activity and GR2 transcript levels, with a significantly high GSH content and high GSH/GSSG ratio of maize seedlings under drought stress (Figs 6 and 7). These results suggest that DCPTA application may prompt the regeneration of GSH by activating the GR enzyme and transcription.

## Conclusion

The results of this research suggest that DCPTA application promoted O_2_·^−^ conversion to H_2_O_2_ by enhancing the SOD activity of seedlings exposed to drought stress. Simultaneously, under drought stress, the up-regulated activity and transcript expression of GR induced by DCPTA maintained the prompt regeneration of GSH from GSSG, favouring stable activity and the transcript expression of MDHAR and DHAR, which promoted the regeneration of AsA from DHA. With increased levels of AsA and up-regulated APX activity and transcript expression, the DCPTA-treated seedlings maintained a high efficiency of H_2_O_2_ quenching. Furthermore, DCPTA application enhanced POD and CAT activities, thus increasing the capacity to scavenge H_2_O_2_. Therefore, the restrained ROS accumulation in DCPTA-treated seedlings resulted in a stronger ability to counter membrane lipid peroxidation and membrane leakiness, maintain normal metabolism, alleviate growth inhibition and confer to maize seedlings enhanced tolerance to PEG-induced drought stress.

## Supporting information

S1 File Supporting information data. This file contains date including the RGR of the shoots and roots.
(XLSX)

S2 File Supporting information data. This file contains date including the generation rate of O_2_·^−^, H_2_O_2_ content, MDA content and EL.
(XLSX)

S3 File Supporting information data. This file contains date including the activities of SOD, POD, CAT, APX, GR, DHAR and MDHAR.
(XLSX)

S4 File Supporting information data. This file contains date including the contents of AsA, DHA, GSH and GSSG, and ratio of AsA/DHA and GSH/GSSG.
(XLSX)

S5 File Supporting information data. This file contains date including the relative expression level of APX1.1, APX1.2, APX2, APX3, APX4, APX5, APX6, APX7, GR1, GR2, DHAR1, DHAR2, DHAR3, MDHAR1, MDHAR2, MDHAR3 and MDHAR4.
(XLSX)
